# Green Streets: Urban Green and Birth Outcomes

**DOI:** 10.3390/ijerph14070771

**Published:** 2017-07-13

**Authors:** Kathryn Abelt, Sara McLafferty

**Affiliations:** Department of Geography & Geographic Information Science, University of Illinois at Urbana-Champaign, 2066 Natural History Building, 1301 W. Green St. Urbana, IL 61801, USA; smclaff@illinois.edu

**Keywords:** greenness, green space, street trees, blue space, birth outcomes, preterm birth, term birthweight, term low birthweight, small for gestational age

## Abstract

Recent scholarship points to a protective association between green space and birth outcomes as well a positive relationship between blue space and wellbeing. We add to this body of literature by exploring the relationship between expectant mothers’ exposure to green and blue spaces and adverse birth outcomes in New York City. The Normalized Difference Vegetation Index (NDVI), the NYC Street Tree Census, and access to major green spaces served as measures of greenness, while proximity to waterfront areas represented access to blue space. Associations between these factors and adverse birth outcomes, including preterm birth, term birthweight, term low birthweight, and small for gestational age, were evaluated via mixed-effects linear and logistic regression models. The analyses were conducted separately for women living in deprived neighborhoods to test for differential effects on mothers in these areas. The results indicate that women in deprived neighborhoods suffer from higher rates adverse birth outcomes and lower levels of residential greenness. In adjusted models, a significant inverse association between nearby street trees and the odds of preterm birth was found for all women. However, we did not identify a consistent significant relationship between adverse birth outcomes and NDVI, access to major green spaces, or waterfront access when individual covariates were taken into account.

## 1. Introduction

For most of our history as a species, humans have lived in and around ‘natural’ environments. However, as a larger share of the world’s population shifts into urban areas, the spaces we inhabit are becoming increasingly built-up and artificial. A great deal of modern scholarship focuses on how decreased contact with natural environments brought on by urbanization impacts human health. Specifically, researchers are interested in the health implications of the presence or absence of ‘green’ spaces and, to a lesser extent, ‘blue’ spaces in cities [1,2].

In the past decade, there have been multiple studies on the relationship between urban green space and birth outcomes [3,4,5,6,7,8,9,10,11,12,13,14,15]. Birth outcomes constitute a major public health issue, as adverse outcomes like term low birthweight, preterm birth, and small for gestational age have been linked to numerous health problems during infancy and later in life [16,17]. In 2013, Kihal-Talantikite, et al. proposed a conceptual framework by which local green space could influence birth outcomes [18]. This framework consists of three main pathways; a psychological pathway, a physiological disruption process, and an environmental pathway. Through these pathways, local green space might improve psychological health, encourage physical activity, promote social capital, and create cleaner, healthier environments for expectant mothers. Higher levels of psychological distress and increased exposure to air pollution among pregnant women have been linked to elevated risk of adverse birth outcomes [19,20], while there is some evidence that physical activity and social capital are protective against such outcomes [21,22]. Since previous studies have established connections between green space and improved psychological health [23], higher rates of physical activity [24], increased social capital [25], and lower levels of air pollution [26], it is possible that one or more of these pathways partially or entirely explains the relationship between urban green space and birth outcomes.

Recent investigations into the association between green space and birth outcomes have demonstrated relatively consistent results [3,4,5,6,7,8,9,10,11,12,13,14,15]. These studies examined a variety of outcomes, but all included at least one outcome related to birthweight and/or gestational age. With two exceptions [10,11], these researchers used the Normalized Difference Vegetation Index (NDVI) as their primary measure of ‘greenness’. In most of the investigations, the analyses were stratified based one or more socioeconomic indicator in order to gauge whether the relationship between birth outcomes and green exposure differed among women of lower socioeconomic status as compared to the overall study population. Assorted covariates representing maternal characteristics and neighborhood-level attributes were also controlled for in each study. The results of these investigations provide support for a protective relationship between green space and birthweight, as a significant positive association between greenness and birthweight-related variables was observed for at least a subset of the population in all studies. The evidence for an association between greenness and birth outcomes related to gestational age was mixed; some researchers identified a significant inverse relationship between green exposure and the risk of preterm birth [5,12,13,14], while others did not [4,6,7,8,10]. For the most part, researchers found stronger associations between greenness and a decreased risk of adverse birth outcomes among women of lower socioeconomic status [4,7,8,9,15].

In this study, we make a unique contribution to the current body of literature by including a variable representing waterfront access (‘blue space’) and a novel green space metric in our analyses. We chose to incorporate a waterfront access variable because, much like exposure to green space, proximity to coastal areas has been linked to better self-reported health [27], lower levels of psychological distress [28], and higher rates of physical activity [29]. This study is also among the first to use street trees as a measure of residential greenness. Researchers have observed a variety of benefits associated with street trees such as reduced rates of asthma in children [30] and lower crime rates [31]. Moreover, street trees represent a more specific, fine-grained measure of local greenness tied to a particular source (trees), as compared to broader metrics like NDVI.

Our research draws on vital statistics from birth records for the year 2000 provided by the New York City Department of Health and Mental Hygiene. Controlling for a range of individual and neighborhood-level covariates, we use multilevel, mixed-effects linear and logistic regression to evaluate the relationship between four adverse birth outcomes–term birthweight, odds of term low birthweight, odds of preterm birth, and odds of small for gestational age—and variables reflecting residential greenness as well as access to major green spaces and waterfront areas. NDVI and the New York City Street Tree Census serve as our two measures of residential greenness, while the presence of major green spaces and waterfront areas within walking distance represent access to green and blue spaces. To test for differential effects on women living in poorer neighborhoods, we conduct analyses separately for mothers residing in economically-deprived census tracts. Based on the results of prior research, we expect to find an inverse relationship between the odds of adverse birth outcomes and the measures of residential greenness, access to major green spaces, and waterfront access. We also predict that this relationship will be stronger for women living in deprived tracts.

## 2. Materials and Methods

### 2.1. Study Area

The study area, New York City, had a population of over eight million in 2000 and an average residential density of >10,000 inhabitants per square kilometer, making it the largest and most densely-populated city in the United States [32]. The city is characterized by ethnic and socioeconomic diversity, with a large foreign-born population and wide disparities in income and wealth. These attributes, along with the city’s famous public green spaces and hundreds of miles of coastline, make it an ideal site to explore the interactions between public health, socioeconomic status, and access to green and blue spaces among a diverse population of urban residents.

### 2.2. Birth Outcomes and Individual Covariates

The data on birth outcomes originate from vital statistics birth records for the year 2000 obtained from the New York City Department of Health and Mental Hygiene. The original dataset consists of all singleton births to mothers who resided within New York City limits (n = 111,754) [33]. For each birth, the dataset contains information on the infant, including birthweight, gestation (weeks), parity, and sex, and the characteristics of the mother such as education, marital status, nativity, race/ethnicity, and risk behaviors. Data are georeferenced by the mother’s census tract of residence. Tracts are census-defined areas that typically contain anywhere from 1200 to 8000 inhabitants. Their physical size can vary greatly depending on local population density [34], but, in New York City, most tracts are small in geographic extent (median = 0.18 km^2^).

For the purposes of this project, the study population was restricted to records without any missing data on birth outcomes or individual-level covariates. All births for which the reported gestation was less than 21 or more than 44 weeks were removed from the analyses, as these records likely represent data entry errors or extreme outliers. Furthermore, mothers living in census tracts with a non-institutionalized population of fewer than 100 residents were not included in the study, leaving a final population size of n = 103,484.

Four separate birth outcomes were analyzed; term birthweight, term low birthweight, preterm birth, and small for gestational age. These variables have been examined in previous studies of birth outcomes and residential greenness, and they are frequently employed as indicators of general neonatal health. Term birthweight was the sole continuous outcome variable, and the analyses were limited to births occurring after 37 or more weeks of gestation. We analyzed term births due to the high degree of correlation between birthweight and gestational age. Term low birthweight was treated as a binary variable, with a value of 1 assigned to infants weighing <2500 g. Preterm birth was also evaluated as a binary variable, wherein births occurring prior to 37 weeks of gestation were assigned a value of 1. The fourth variable, small for gestational age (SGA), refers to infants that fall into the bottom decile of birthweight stratified by sex and gestational age in weeks. In this study, the deciles were computed based on birthweights for the overall study population. SGA infants were assigned a value of 1.

Maternal and infant characteristics that have been demonstrated to affect birth outcomes were controlled for in the analyses. Maternal race, world region of birth, high school education, marital status, and receipt of Medicaid were represented as binary and categorical variables. Medicaid is the U.S. government’s program that provides health care coverage for low-income adults and children. Maternal age was analyzed as a continuous variable. Maternal smoking and alcohol use, both behaviors known to impact birth outcomes, were also treated as binary variables, with instances in which the mother did not report any smoking or alcohol use during pregnancy assigned a value of 0. In analyses of term birthweight and odds of term low birthweight, the reported length of gestation (in weeks) was incorporated as a continuous variable. The infant’s sex and season of birth were controlled for in all the statistical tests. All covariates were selected based on demonstrated significant relationships to birth outcomes in chi-squared tests or simple linear and logistic regression models.

### 2.3. Measures of Green and Blue Space

**Figure 1:**
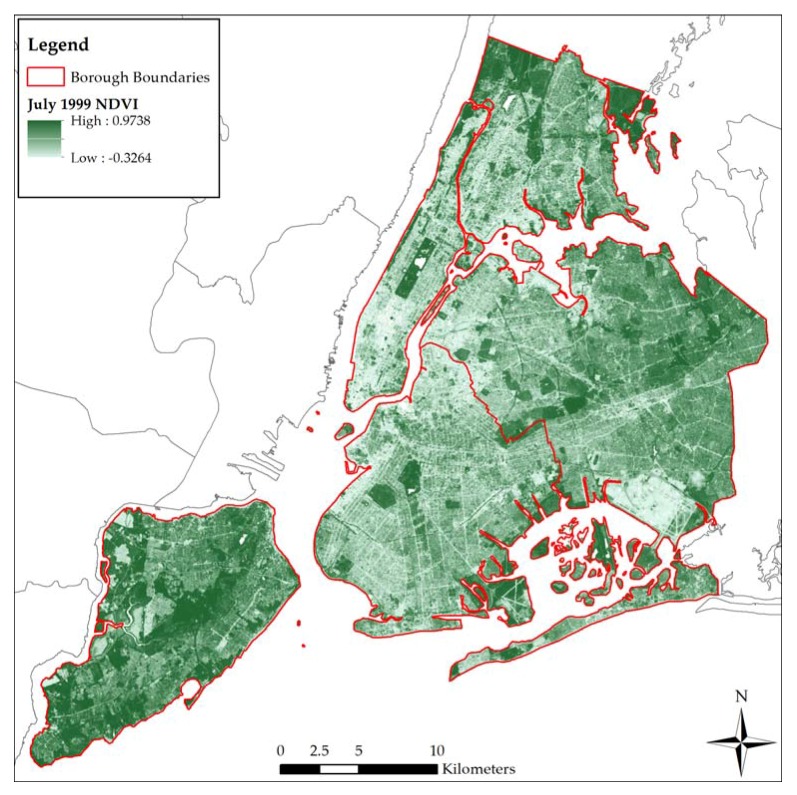
Distribution of Normalized Difference Vegetation Index (NDVI) values in New York City.

The Normalized Difference Vegetation Index, or NDVI, served as this study’s primary metric for residential greenness. Since the pigment in plant leaves absorbs visible light (VIS) but reflects near-infrared light (NIR), the density of vegetation in an area can be approximated using a mathematical formula where (NIR − VIS) ÷ (NIR + VIS) = NDVI. NDVI values range from −1 to 1, with negative values and values around zero representing little to no vegetation, while values closer to 1 correspond to high-density plant life [35]. The NDVI data for this project were obtained from the U.S. Geological Survey’s (USGS) EarthExplorer website. Pre-calculated land surface reflectance NDVI data originating from a single Landsat 5 TM 30 × 30 m resolution satellite image were downloaded. We selected this image because it contained minimal (<10%) cloud contamination, encompassed the whole of the study area, and was captured during the Summer (specifically on 6 July 1999), which is generally the ‘greenest’ portion of the year in the northern hemisphere. After importing the image into the Environmental Systems Research Institute’s ArcGIS 10.4 (ESRI, Redlands, CA, USA), the pixels representing water were removed using a CFMask raster layer from the EarthExplorer website. Figure 1 illustrates the distribution of NDVI values throughout the city.

Mean NDVI values were calculated for 100 m, 250 m, and 500 m circular buffers around the population-weighted centroid of each mother’s census tract of residence. These three buffer sizes were selected to represent varying distances from the mother’s residence, ranging from the space immediately surrounding her home to areas a short walk away. All of these buffer sizes have been employed in previous studies establishing a link between NDVI and birth outcomes [3].

**Figure 2:**
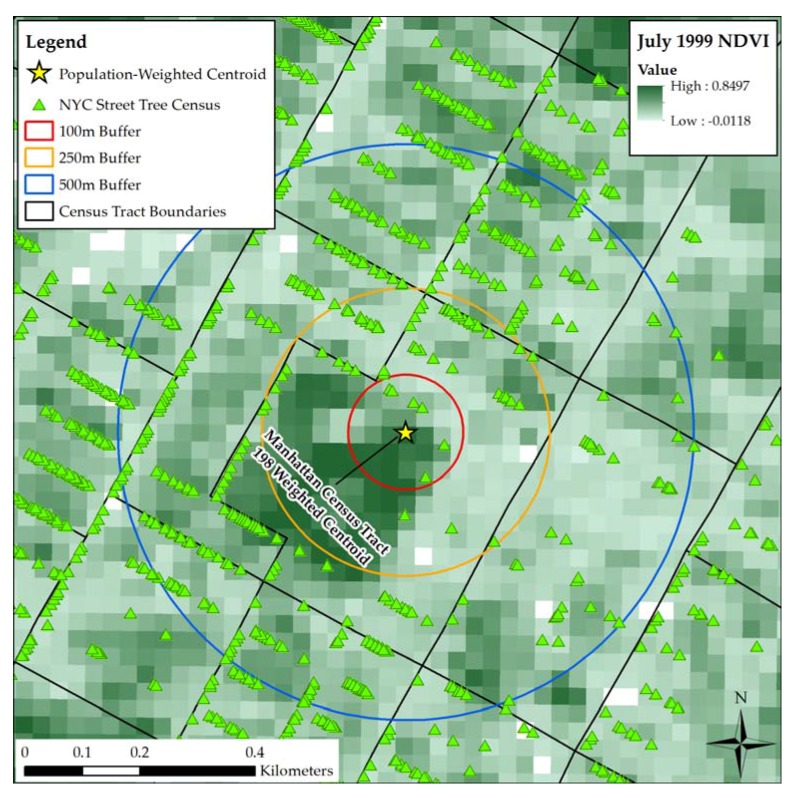
Example measures of greenness exposure.

The 2005–2006 New York City Street Tree Census acted as an additional measure of residential greenness. Overseen by the New York City Parks Department, this census was conducted over the span of two summers by volunteers, who recorded the location, size, species, and condition of each tree growing alongside a city street. They identified 592,130 trees in total [36]. The recorded locations of the trees in decimal degrees were used to generate a point file in ArcMap, and the trees were joined to tract buffers to calculate the numbers that fell within 100 m, 250 m, and 500 m of each tract’s population-weighted centroid (Figure 2).

Proximity to major green spaces like parks and other large, vegetated areas was also considered. Data on these spaces came from the MapPLUTO 16v1 dataset developed by the NYC Department of City Planning (DCP). This dataset contains geographic and land use information compiled by local government agencies and aggregated at the tax lot level [37]. All tax lots designated with the numeric land use code corresponding to ‘Open Space and Outdoor Recreation’ were considered green spaces. Green spaces less than 5000 square meters in area were excluded from the analyses, which was in keeping with a public open space indicator developed by the European Commission [38] and employed in previous research on green space and birth outcomes [4].

ArcGIS’s network analyst extension was used to measure access to these spaces based on an 800 m street network buffer around each population-weighted census tract centroid. Designed to represent a half-mile walking distance, the buffer size was selected based on park accessibility goals set forward by several major U.S. cities [39]. The weighted tract centroids with service areas that intersected with one or more major green spaces were assigned a value of 1, indicating adequate spatial access to major green space(s).

**Figure 3:**
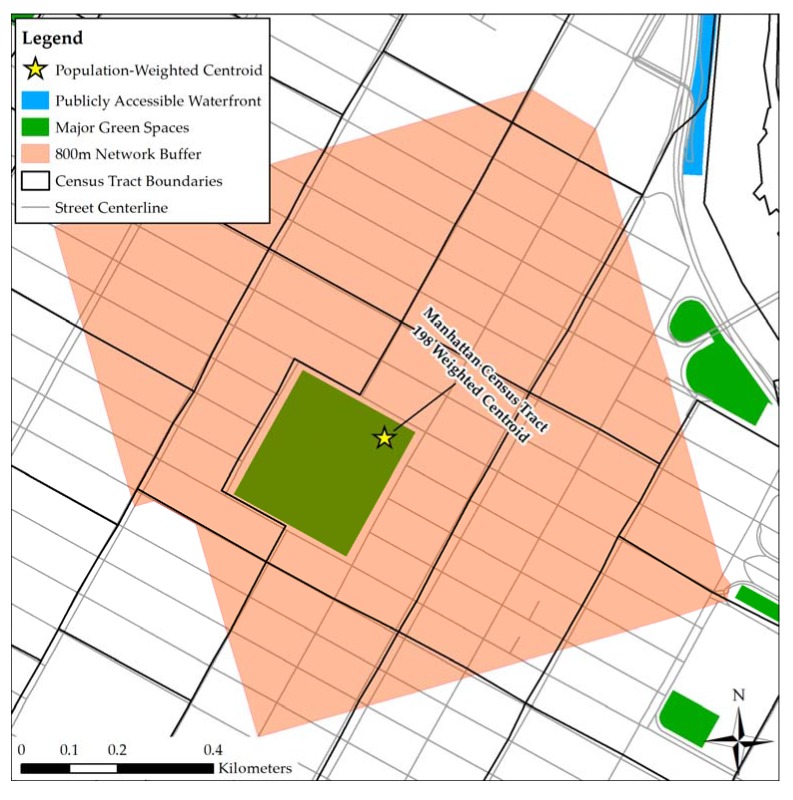
Example measures of access to major green spaces and waterfront areas.

Access to nearby waterfront locations served as the measure of exposure to blue space. This variable was estimated using the NYC Waterfront Parks shapefile and the Publicly Accessible Waterfront Spaces (PAWS) shapefiles that represent waterfront spaces that people can access [40]. Using the street-network buffer service areas that were previously calculated, we determined if each weighted census tract centroid fell within an 800 m path distance of publicly accessible waterfront. The centroids that met this criterion were assigned a value of 1. Figure 3 provides an example of how access to green and blue spaces was quantified.

### 2.4. Neighborhood Covariates

To control for additional neighborhood characteristics that might influence birth outcomes, we incorporated several potentially confounding area-level variables. The distance to the nearest major road served as a substitute measure of exposure to traffic-related air and noise pollution. Data on major roads originated from the U.S. Census Bureau’s 2010 Topologically Integrated Geographic Encoding and Referencing (TIGER) primary and secondary roads shapefile. The distance to the nearest tax lot designated as ‘Industrial and Manufacturing’ in the MapPLUTO land use dataset was employed as a surrogate metric for local air pollution due to the lack of access to detailed air pollution data for the study period. Straight-line distances from tract centroids to both of these facilities were calculated in ArcGIS. Previous studies have used distance to the nearest major road as a substitute for traffic-related air pollution [5,7], while others have incorporated proximity to industrial land use into land use regression models to estimate particulate matter concentration in New York City [41]. As in prior studies [10,15], population density was employed as a tract-level covariate to represent differences in residential contexts across the city, from high-rise areas of Manhattan to the suburban landscapes of Staten Island, which may be associated with birth outcomes.

To control for neighborhood socioeconomic status, a tract-level deprivation index was created based on eight variables; the percentage of female-headed households with children under 18 and no husband present; the percentage of households receiving public assistance income; the percentage of households whose yearly income was less than $35,000; the percentage of individuals living below the poverty line; the percentage of individuals over 16 years old who were unemployed; the percentage of employed individuals over 16 years old who worked in management or professional occupations; the percentage of adults over 25 years old with less than a 12th grade education; and the percentage of occupied housing units with more than one occupant per room. These indicators came from a standardized neighborhood deprivation index developed by Messer et al. [42]. Principal components analysis was used to summarize the common variation in these correlated variables, and the first principal component, which accounted for approximately 68% of the total variance, was extracted as a deprivation index. The scores on the index ranged from −4.32 to 7.05, with lower values corresponding to less deprived tracts and higher values corresponding to more deprived tracts.

### 2.5. Statistical Methods

Multilevel mixed-effects linear and logistic regression was used to model the associations between tract and individual-level variables and birth outcomes. Each birth outcome served as the dependent variable in a series of two-level multilevel models. Individual covariates comprised the first-level variables, while the second-level variables included measures of greenness, access to waterfront and major green spaces, and environmental and socioeconomic covariates. Altogether there were 103,484 individual records nested within 2132 tracts. The number of individual records associated with each tract ranged from one to 263, with a mean of 48.5.

We used Stata’s xtmixed and xtmelogit commands to conduct mixed-effects linear and logistic regression. We first ran unadjusted models for each birth outcome, with each measure of greenness and blue space serving as the sole independent variables. Then, fully adjusted models containing the aforementioned first and second-level variables were run, with separate analyses conducted for each buffer size. All models included the census tract identifier as a random effect to account for potential neighborhood-level variation not captured by any of the tract-level variables. In order to gauge the differential effects of greenness by neighborhood deprivation, analyses were conducted separately for women living in deprived tracts. A tract was classified as deprived if its neighborhood deprivation index value was above the median.

For all birth outcomes, two sets of unadjusted models were run, with one set employing measures of residential greenness (mean NDVI and street tree count) as the independent variables and another with access to major green spaces and waterfront areas serving as the independent variables. Unadjusted and adjusted models incorporating measures of greenness were run separately at each buffer size in order to minimize collinearity.

## 3. Results

### 3.1. Descriptive Statistics

Adverse birth outcomes comprised a minority of birth records in the study population. Just under 3% of term-born infants were classified as low birthweight, while 11.13% and 10.15% of all neonates were classified as preterm or small for gestational age, respectively (Table 1). The rates of all three binary adverse birth outcomes were higher among women living in deprived tracts, and the mean term birthweight of infants born to mothers in these tracts was lower than that of infants born to women in non-deprived tracts. Mothers in these tracts were generally younger and less likely to be married or have a high school diploma (Table 2). Women living in deprived tracts also engaged in risk behaviors like alcohol and tobacco use during pregnancy at higher rates than mothers residing in non-deprived tracts.

Deprived tracts were, on average, less ‘green’ than non-deprived tracts in terms of both mean NDVI values and street tree counts at all buffer levels (Table 3). These tracts were also more densely populated and tended to be closer to potential disamenities like major roads and industrial land use. However, deprived tracts were more likely to fall within an 800 m street network distance of major green spaces and publicly accessible waterfront areas than non-deprived tracts. The majority of mothers were classified as having access to a nearby major green space, while just over a quarter of mothers were classified as having access to a publicly accessible waterfront.

### 3.2. Mixed-Effects Linear Regression: Term Birthweight

Unadjusted models for the entire study population showed a statistically significant (*p* < 0.05) positive relationship between street tree counts and term birthweight at all buffer sizes (Table 4). Mean NDVI also had a positive association with term birthweight in these models; however, this association was not statistically significant at any buffer level. A significant inverse relationship between access to major green spaces and term birthweight was observed in the unadjusted model for the overall study population (Table S1), while no significant association was found between term birthweight and access to waterfront locations (Table S2).

In fully adjusted models for the overall study population, no significant relationship between either measure of greenness and term birthweight was observed (Table 4), nor were there any significant associations between term birthweight and access to major green spaces or waterfront locations. Apart from population density, which showed a significant inverse relationship with term birthweight in these models, none of the neighborhood covariates were significantly associated with term birthweight.

Like the unadjusted models for the entire study population, the unadjusted models restricted to women in deprived tracts revealed a statistically significant positive link between street tree counts and term birthweight (Table 4). However, these models also demonstrated a significant inverse relationship between mean NDVI and term birthweight at all buffer sizes. Associations between term birthweight and access to major green spaces and waterfront locations were non-significant (Table S1). In the fully adjusted models restricted to mothers in deprived tracts, no statistically significant relationship was identified between either measure of greenness, access to major green spaces and waterfront locations, or any of the neighborhood covariates.

### 3.3. Mixed-Effects Logistic Regression: Odds of Term Low Birthweight

In the unadjusted models for the overall study population, the street tree count showed a significant inverse relationship with the odds of term low birthweight. Mean NDVI, access to major green spaces, and waterfront access were not significantly associated with term low birthweight in these models (Table 5 and Tables S1 and S2). In the fully adjusted models for the overall study population, by contrast, there was no statistically significant relationship between term low birthweight and both measures of residential greenness at any buffer size (Table 5). Additionally, no significant relationship was observed between term low birthweight and access to major green spaces, waterfront access, or any of the neighborhood-level covariates.

Among the women living deprived tracts, the results of the unadjusted models for low term birthweight differed slightly from those of the overall study population. The street tree count was still inversely associated with the odds of term low birthweight, but this relationship was only significant at the 500 m buffer level (Table 5). Conversely, mean NDVI demonstrated a significant positive relationship with the odds of term low birthweight at all buffer sizes. There was no significant relationship between low term birthweight and access to major green spaces or public waterfront areas (Tables S1 and S2). As was the case in the fully adjusted models for the overall study population, none of the variables of interest were significantly related to low term birthweight in fully adjusted models (Table 5). 

### 3.4. Mixed-Effects Logistic Regression: Odds of Preterm Birth

In the unadjusted models representing the entire study population, both the street tree counts and the mean NDVI values were inversely associated with the odds of preterm birth at all buffer sizes; however, this relationship was only statistically significant for the street tree counts (Table 6). Access to major green spaces showed a significant positive association with the odds of preterm birth in the unadjusted model (Table S1), while no significant relationship was found between waterfront access and the odds of preterm birth (Table S2).

In the fully adjusted models for the overall study population, the street tree counts at all buffer levels had a significant inverse relationship with the odds of preterm birth (Table 6). The mean NDVI was also inversely associated with the odds of preterm birth, but this relationship was only marginally (*p* < 0.1) significant at the 100 m buffer level and not at all significant within the 250 m and 500 m buffers. Tract-level neighborhood deprivation was significantly correlated with heightened odds of preterm birth in all models, while tract population density showed a significant negative association with preterm birth. Access to major green spaces and waterfront areas was not significantly related to the odds of preterm birth in these models nor were any of the other neighborhood-level covariates.

In the unadjusted models restricted to mothers living in deprived tracts, the street tree count at all buffer levels showed a significant inverse relationship with the odds of preterm birth (Table 6). Mean NDVI was positively associated with the odds of preterm birth at all buffer sizes, but these relationships were not significant. As was the case in the unadjusted model for the overall study population, access to major green space was positively associated with the odds of preterm birth to a significant degree (Table S1), while no significant association was identified between preterm birth and waterfront access (Table S2).

In the fully adjusted models restricted to deprived tracts, the street tree count showed a marginally significant inverse association with the odds of preterm birth within a 100 m buffer (Table 6) and fully significant inverse associations in the models for 250 m and 500 m buffers. The effect of mean NDVI was not at all significant. Tract-level deprivation was no longer significant in any of the adjusted models, and population density only retained its significance in the model incorporating measures of greenness within a 100 m buffer. Neither access to major green spaces nor waterfront access was significantly related to the odds of preterm birth in any of these models.

### 3.5. Mixed-Effects Logistic Regression: Odds of Small for Gestational Age

In the unadjusted models for the overall study population, the street tree count had a significant inverse relationship with the odds of SGA at all buffer levels (Table 7). Mean NDVI showed a positive association with odds of SGA in these models, but this relationship was not significant at any buffer size. In the fully adjusted models for the overall study population, neither measure of greenness had a statistically significant relationship with the odds of SGA at any buffer size. Access to major green spaces, waterfront access, and neighborhood covariates were not significantly associated with SGA in these models either.

Among women living in deprived tracts, there was a statistically significant positive relationship between mean NDVI and the odds of SGA in the unadjusted models at all buffer levels (Table 7). Access to major green space was also significantly associated with heightened odds of SGA in the unadjusted model for this subset of the population (Table S1). Meanwhile, the street tree count showed a negative association with the odds of SGA at all buffer sizes, but this effect was only significant within the 500 m buffer.

The fully adjusted models restricted to deprived tracts differed quite noticeably from the fully adjusted models for the overall population. While mean NDVI was not significantly associated with the odds of SGA at the 100 m buffer level (Table 7), it showed a statistically significant positive relationship with the odds of SGA within the 250 and 500 m buffers. By contrast, street tree counts, access to major green spaces, waterfront access, and other neighborhood covariates were not significantly associated with the odds of SGA at any buffer size in these models.

## 4. Discussion

### 4.1. Environmental Factors and Birth Outcomes

This study uncovered partial support for an association between residential greenness and birth outcomes. The most notable finding was evidence of an inverse relationship between local street trees and increased odds of preterm birth. These results point to potential health benefits for expectant mothers associated with street trees near their homes. In comparison, no consistent significant associations were observed for mean NDVI, access to major green spaces, or waterfront access.

These results differ from the majority of the findings from prior research on the relationship between residential greenness and variables related to birthweight. In nearly all studies, researchers identified a statistically significant relationship between measures of residential greenness and birthweight outcomes for at least a subset of the population in their final models [6,7,8,9,11,12,13,14,15]. Only Casey et al. [5] also failed to identify a significant relationship between greenness and term birthweight.

In this study, the fact that the associations between measures of greenness and variables related to term birthweight lost their significance when individual covariates were added to the models indicates that individual or neighborhood-level covariates correlated with greenness might have a greater impact on term birthweight than greenness itself. Since there was no statistically significant relationship between neighborhood covariates and the odds of term low birthweight in any of the models, it is also possible that there was a selection effect wherein women at lesser risk of delivering a term-born, lower birthweight infant tended to live in greener, less deprived neighborhoods.

In the unadjusted and adjusted models, we identified a significant inverse relationship between street tree counts at all buffer levels and the odds of preterm birth. The results of these analyses indicate that localized forms of greenness like street trees may have a protective effect against preterm birth. The consistency of results for street trees as compared to NDVI suggests that fine-grained, street-level vegetation is more beneficial for reducing the likelihood of preterm birth than a neighborhood’s raw vegetation density. In addition, the similarity of the relationship between street trees and the odds of preterm birth among mothers in deprived tracts and mothers in the overall study population suggests that nearby street trees could be protective against preterm birth for pregnant women regardless of their socioeconomic status.

The results of mixed-effects regression models for SGA offered less support for our hypothesis. While mean NDVI was not significantly related to the odds of SGA in the fully adjusted models for the overall population, among women in deprived tracts, mean NDVI within 250 m and 500 m buffers had a significant positive relationship with the odds of SGA. This outcome is inconsistent with prior research on the association between residential greenness and SGA. Of the previous studies that examined this relationship, three uncovered a significant inverse association between greenness and the odds of SGA [5,10,13], and none of the studies identified a significant positive relationship between greenness and the odds of SGA for any subset of the population.

It might be the case that women in the city’s deprived neighborhoods perceive highly vegetated areas as unsafe and/or poorly maintained. Unsafe, unattractive green spaces could function as ‘disamenities’ for local pregnant women, causing them to feel distressed and anxious and potentially discouraging them from visiting the spaces to engage in outdoor physical activity and social interaction. Additional research is necessary to clarify the relationship between expectant mothers’ perceptions of local green space and adverse pregnancy outcomes like SGA for women living in deprived neighborhoods.

### 4.2. Implications

Our results represent an important addition to the growing body of research on green space and birth outcomes. No previous study on this subject has employed a citywide street tree census as a measure of greenness, making this project methodologically unique. The significant inverse association found between street trees and the odds of preterm birth highlights the benefits of urban forestry initiatives, while the lack of greenness identified in deprived neighborhoods calls attention to patterns of environmental injustice facing expectant mothers of lower socioeconomic status.

This study’s findings could provide useful guidance for policymakers seeking to improve the health and wellbeing of urban residents. First and foremost, the significant associations between local street trees and reduced odds of preterm birth identified in these analyses underscore the importance of the tree canopy in major cities. Neonates born prematurely often suffer from health complications throughout infancy, putting emotional and financial strain on their families [17]. There is also evidence that infants born prematurely continue to experience cognitive and behavioral difficulties later in childhood [43], which places an additional burden on communities. Increasing the number of street trees in a neighborhood as well as investing in the maintenance of existing trees are simple and inexpensive interventions that could help ameliorate a serious public health problem. Furthermore, planting and maintaining street trees in economically disadvantaged neighborhoods has the potential to reduce environmental injustice. In this study, we observed that women living in deprived census tracts generally experienced lower levels of exposure to street trees, and previous research provides additional evidence for this phenomenon [44]. Prioritizing urban forestry initiatives in economically disadvantaged areas might help alleviate the burden that adverse birth outcomes place on women and families living in these neighborhoods.

For the most part, our analyses did not reveal a consistent significant relationship between adverse birth outcomes and residential greenness as measured by NDVI or access to major green spaces. Since there is evidence that the quality of neighborhood green space may be more important than the quantity in terms of benefitting the health and wellbeing of nearby residents [45,46], it is possible that this lack of significance can be attributed to the fact that we were unable to take the quality of local green spaces into account. Moreover, prior research has demonstrated that members of marginalized communities in New York City already have sufficient ‘access’ to green spaces when ‘access’ is defined as living within a reasonable walking distance of a park [47]; however, these same communities are also more likely to suffer from higher rates of neighborhood disamenities like violent crime, traffic hazards, and pollution [48]. These disamenities could serve to both degrade the perceived safety and/or aesthetic value of local green spaces and discourage residents from leaving their homes in order to visit those spaces. For this reason, policymakers should prioritize efforts to make deprived neighborhoods safer and healthier places to live over efforts to make them ‘greener’ in terms of sheer vegetation density.

### 4.3. Limitations and Recommendations for Future Research

This work has several limitations. The measures of exposure to residential greenness and access to major green spaces and waterfront areas employed here are limited in the sense that they may not reflect expectant mothers’ actual exposure and access to these environments. For instance, it is possible that some of the mothers moved during pregnancy, thus causing their exposure and access to green and blue spaces to differ from this study’s measurements. Similarly, our measures of access to major green spaces and waterfront areas do not reflect how often and/or for what purpose expectant mothers visit these spaces. We were also unable to incorporate measures of the quality of residential greenness, major green spaces, and publicly accessible waterfronts into our analyses. Finally, we were unable to assess the potential pathways proposed by Kihal-Talantikite et al. [18] by which green space might influence birth outcomes. Of these three pathways, only one is partially accounted for in this study through proxy variables for exposure to air pollution and traffic-related noise. Due to the limited information contained in vital statistics birth records, we were unable to control for maternal physical activity, social capital, or psychological wellbeing.

We propose several recommendations for future studies that could mitigate these limitations. First, prospective researchers should attempt to elucidate the pathways linking green and blue spaces to birth outcomes. Ideally, data on maternal physical activity, social capital, and psychological health would be collected throughout pregnancy via questionnaires and/or interviews. Improvements to the availability and accuracy of fine-scale environmental data will also provide researchers with better approximations of exposure to air pollution and other relevant ecological factors. Second, future researchers should take the quality of local green and blue spaces into account. Subjective measures of the quality of these spaces could be obtained through interviews with women in the study population, with researchers gauging their perceptions of the safety, cleanliness, aesthetic value, and overall desirability of these spaces. Trained auditors could also be deployed to provide a more objective measure of the quality of these spaces. Third, future studies should incorporate measures of exposure to greenness beyond just a residential context. Data on expectant mothers’ daily activity spaces could be collected via travel diaries and/or GPS tracking. Using these methods to gather data on the mothers’ patterns of movement would provide more accurate information on the frequency, duration, and purpose of participants’ exposures to local green and blue spaces. Finally, prospective researchers should consider using street trees as a measure of green exposure. The results of this study indicate the potential health benefits of street trees for expectant mothers and their newborn infants. A better understanding of the relationship between street trees and birth outcomes will provide planners and policymakers in urban areas with useful guidance in their efforts to design neighborhoods that promote residents’ health and wellbeing.

## 5. Conclusions

The results of our analyses point to a significant association between street trees surrounding the home and reduced odds of preterm birth. However, apart from a positive relationship between the odds of SGA and mean NDVI at certain buffer sizes in deprived tracts, no consistent significant relationship was identified between adverse birth outcomes and access to major green spaces, waterfront access, or NDVI. These findings suggest that exposure to fine-scale, street-level green space like street trees might be more consistently beneficial for certain facets of neonatal health than other varieties of local green space. Furthermore, our findings highlight the importance of urban forestry initiatives. A better understanding of the health impacts of street trees will help researchers, planners, and policymakers build neighborhoods that are more conducive to their residents’ wellbeing.

## Figures and Tables

**Table 1:** Birth outcomes and infant covariates.

Birth Outcome	Study Population (n = 103,484)	Deprived Tracts (n = 51,787)	Non-Deprived Tracts (n = 51,697)
**Gestational age in weeks** **(mean, *std. dev.*)**	38.78, *2.46*	38.71, *2.62*	38.84, *2.28*
**Term birthweight in grams** **(mean, *std. dev.*)**	3379.51, *477.01*	3363.46, *482.72*	3395.11, *470.88*
**Term low birthweight (%)**			
Yes	2.91	3.21	2.62
No	97.09	96.79	97.38
**Preterm birth (%)**			
Yes	11.13	12.49	9.78
No	88.87	87.51	90.22
**Small for gestational age (%)**			
Yes	10.15	10.91	9.38
No	89.85	89.09	90.62
**Sex (%)**			
Male	51.35	51.34	51.36
Female	48.65	48.66	48.64
**Season of birth (%)**			
Spring	24.24	23.57	24.92
Summer	26.42	26.62	26.22
Fall	25.55	25.69	25.42
Winter	23.78	24.12	23.44

**Table 2:** Maternal covariates.

Covariate	Study Population (n = 103,484)	Deprived Tracts (n = 51,787)	Non-Deprived Tracts (n = 51,697)
**Age (mean, *std. dev.*)**	28.04, *6.34*	26.58, *6.30*	29.49, *6.05*
**Finished high school (%)**			
Yes	74.46	63.42	85.51
No	25.54	36.58	14.49
**Married (%)**			
Yes	52.85	35.84	69.88
No	47.15	64.16	30.12
**Medicaid recipient (%)**			
Yes	55.06	72.8	37.28
No	44.94	27.2	62.72
**Tobacco use while pregnant (%)**			
Yes	3.44	4.23	2.65
No	96.56	95.77	97.35
**Alcohol use while pregnant (%)**			
Yes	0.22	0.29	0.14
No	99.78	99.71	99.86
**Race (%)**			
White	54.29	47.05	61.54
Black	32.98	44.46	21.48
East Asian	6.37	5.1	7.64
South Asian	4.33	2.15	6.51
Other Asian/Pacific Islander	1.54	0.87	2.21
Other/Unknown	0.49	0.36	0.62
**Birth region (%)**			
U.S. and Canada	45.06	43.14	46.98
Caribbean	18.7	26	11.38
Mexico and Central America	8.19	11.05	5.32
South America	6.77	6.16	7.37
Western Europe	1.77	0.5	3.05
Eastern Europe	3	0.93	5.08
Sub-Saharan Africa	2.55	3.6	1.49
Middle East and North Africa	2.38	0.88	3.88
Central Asia	0.52	0.14	0.9
South Asia	3.82	1.93	5.72
East Asia	5.77	4.78	6.75
Southeast Asia	1.34	0.83	1.86
Other/Unknown	0.14	0.06	0.22

**Table 3:** Measures of greenness and neighborhood covariates.

Neighborhood-Level Variable	Study Population (n = 103,484)	Deprived Tracts (n = 51,787)	Non-Deprived Tracts (n = 51,697)
**Average NDVI (mean, *std. dev.*)**			
100 m buffer	0.28, *0.11*	0.25, *0.10*	0.31, *0.12*
250 m buffer	0.28, *0.10*	0.26, *0.07*	0.31, *0.11*
500 m buffer	0.29, *0.09*	0.27, *0.06*	0.32, *0.10*
**Street tree count (mean, *std. dev.*)**			
100 m buffer	32.77, *22.58*	26.87, *18.84*	38.68, *24.40*
250 m buffer	201.92, *104.55*	163.97, *79.97*	239.94, *112.18*
500 m buffer	740.24, *329.08*	608.98, *254.82*	871.73, *342.32*
**Distance to industrial land use in meters (mean, *std. dev.*)**	343.66, *311.74*	253.27, *182.19*	434.21, *380.66*
**Distance to nearest major road in meters (mean, *std. dev.*)**	985.13, *820.39*	900.36, *728.84*	1070.05, *894.84*
**Neighborhood deprivation index (mean, *std. dev.*)**	0.69, *2.44*	2.74, *1.36*	−1.35, *1.33*
**Population density per sq. kilometer (mean, *std. dev.*)**	26,011.19, *16,600.86*	31,761.42, *15,727.65*	20,250.95, *15,413.25*
**800 m from major green space (%)**			
Yes	84.97	88.42	81.52
No	15.03	11.58	18.48
**800 m from publicly accessible waterfront (%)**			
Yes	25.44	27.76	23.11
No	74.56	72.24	76.89

**Table 4:** Mixed-effects linear regression results for term birthweight.

Term Births in Study Population (n = 91,963)			Term Births in Deprived Tracts (n = 45,321)		
Model	Coef.	95% CI	Model	Coef.	95% CI
**Mean NDVI (100 m buffer)**					
Adjusted	5.9547	−27.4096–39.3189	Adjusted	−22.0388	−72.9623–28.8846
Unadjusted	6.5678	−25.3591–38.4946	Unadjusted	−97.2777 *	−151.1224–−43.4330
**Mean NDVI (250 m buffer)**					
Adjusted	9.0173	−32.3941–50.4288	Adjusted	−65.6636	−133.2686–1.9413
Unadjusted	3.9892	−33.0152–40.9937	Unadjusted	−159.8797 *	−228.5841–−91.1753
**Mean NDVI (500 m buffer)**					
Adjusted	31.3106	−14.1890–76.8102	Adjusted	−47.0084	−126.0089–31.9921
Unadjusted	17.6882	−22.5183–57.8947	Unadjusted	−141.7667 *	−222.1642–−61.3692
**Street tree count (100 m buffer)**					
Adjusted	−0.0112	−0.1608–0.1385	Adjusted	0.0086	−0.2288–0.2460
Unadjusted	0.5221 *	0.3616–0.6826	Unadjusted	0.3419 *	0.0671–0.6167
**Street tree count (250 m buffer)**					
Adjusted	0.0109	−0.0255–0.0473	Adjusted	0.0031	−0.0566–0.0628
Unadjusted	0.1597 *	0.1253–0.1941	Unadjusted	0.1178 *	0.0540–0.1816
**Street tree count (500 m buffer)**					
Adjusted	0.0078	−0.0043–0.0198	Adjusted	0.0087	−0.0115–0.0289
Unadjusted	0.0523 *	0.0413–0.0632	Unadjusted	0.0453 *	0.0254–0.0653

* Denotes significance. The adjusted models control for maternal age, race, region of birth, education, Medicaid status, marital status, tobacco use during pregnancy, and alcohol use during pregnancy; the infant’s gestational age, sex, and season of birth; the distance to industrial land use; the distance to the nearest major road; neighborhood deprivation; population density; access to major green space; and access to publicly accessible waterfront areas.

**Table 5:** Mixed-effects logistic regression results for the odds of term low birthweight.

Term Births in Study Population (n = 91,963)			Term Births in Deprived Tracts (n = 45,321)		
Model	OR	95% CI	Model	OR	95% CI
**Mean NDVI (100 m buffer)**					
Adjusted	0.8971	0.5866–1.3719	Adjusted	1.0009	0.9978–1.0039
Unadjusted	1.1054	0.7761–1.5744	Unadjusted	2.2958 *	1.2878–4.093
**Mean NDVI (250 m buffer)**					
Adjusted	1.0751	0.6331–1.8258	Adjusted	1.5767	0.6823–3.6437
Unadjusted	1.2547	0.8306–1.8956	Unadjusted	3.4033 *	1.6151–7.1712
**Mean NDVI (500 m buffer)**					
Adjusted	1.0847	0.6030–1.9512	Adjusted	1.4897	0.5607–3.9583
Unadjusted	1.2335	0.7842–1.9401	Unadjusted	3.2889 *	1.3832–7.8205
**Street tree count (100 m buffer)**					
Adjusted	1.0002	0.9982–1.0021	Adjusted	1.0009	0.9978–1.0039
Unadjusted	0.9965 *	0.9946–0.9983	Unadjusted	0.9988	0.9957–1.0018
**Street tree count (250 m buffer)**					
Adjusted	1.0000	0.9995–1.0005	Adjusted	1.0002	0.9995–1.0010
Unadjusted	0.9989 *	0.9985–0.9993	Unadjusted	0.9995	0.9988–1.0002
**Street tree count (500 m buffer)**					
Adjusted	0.9999	0.9998–1.0001	Adjusted	0.9999	0.9997–1.0002
Unadjusted	0.9996 *	0.9995–0.9998	Unadjusted	0.9997 *	0.9995–0.9999

* Denotes significance. The adjusted models control for maternal age, race, region of birth, education, Medicaid status, marital status, tobacco use during pregnancy, and alcohol use during pregnancy; the infant’s gestational age, sex, and season of birth; the distance to industrial land use; the distance to the nearest major road; neighborhood deprivation; population density; access to major green space; and access to publicly accessible waterfront areas.

**Table 6:** Mixed-effects logistic regression results for the odds of preterm birth.

Study Population (n = 103,484)			Deprived Tracts (n = 51,787)		
Model	OR	95% CI	Model	OR	95% CI
**Mean NDVI (100 m Buffer)**					
Adjusted	0.8192	0.6571–1.0213	Adjusted	0.74646	0.5327–1.0460
Unadjusted	0.9256	0.7494–1.1432	Unadjusted	1.3161	0.9281–1.8663
**Mean NDVI (250 m buffer)**					
Adjusted	0.8406	0.6378–1.1078	Adjusted	0.7187	0.4580–1.1277
Unadjusted	0.934	0.7311–1.1932	Unadjusted	1.5019	0.9565–2.3584
**Mean NDVI (500 m buffer)**					
Adjusted	0.9109	0.6705–1.2373	Adjusted	0.83	0.4887–1.4097
Unadjusted	0.9219	0.7060–1.2039	Unadjusted	1.6211	0.9569–2.7465
**Street tree count (100 m buffer)**					
Adjusted	0.9989 *	0.9978–0.9999	Adjusted	0.9984	0.9968–1.0001
Unadjusted	0.9954 *	0.9944–0.9965	Unadjusted	0.9970 *	0.9951–0.9988
**Street tree count (250 m buffer)**					
Adjusted	0.9998 *	0.9995–1	Adjusted	0.9996 *	0.9992–0.9999
Unadjusted	0.9987 *	0.9985–0.9990	Unadjusted	0.9992 *	0.9987–0.9996
**Street tree count (500 m buffer)**					
Adjusted	0.9999 *	0.9998–0.9999	Adjusted	0.9998 *	0.9997–0.9999
Unadjusted	0.9996 *	0.9995–0.9996	Unadjusted	0.9997 *	0.9996–0.9999

* Denotes significance. The adjusted models control for maternal age, race, region of birth, education, Medicaid status, marital status, tobacco use during pregnancy, and alcohol use during pregnancy; the infant’s gestational age, sex, and season of birth; the distance to industrial land use; the distance to the nearest major road; neighborhood deprivation; population density; access to major green space; and access to publicly accessible waterfront areas.

**Table 7:** Mixed-effects logistic regression results for small for gestational age.

Study Population (n = 103,484)			Deprived Tracts (n = 51,787)		
Model	OR	95% CI	Model	OR	95% CI
**Mean NDVI (100 m buffer)**					
Adjusted	1.0034	0.8043–1.2519	Adjusted	1.2182	0.8794–1.6877
Unadjusted	1.0294	0.8474–1.2504	Unadjusted	1.7742 *	1.3031–2.4156
**Mean NDVI (250 m buffer)**					
Adjusted	1.1668	0.8854–1.5377	Adjusted	1.7084 *	1.1062–2.6384
Unadjusted	1.119	0.8923–1.4031	Unadjusted	2.5941 *	1.7452–3.8559
**Mean NDVI (500 m buffer)**					
Adjusted	1.2881	0.9482–1.7498	Adjusted	2.0172 *	1.2149–3.3493
Unadjusted	1.1186	0.8734–1.4326	Unadjusted	2.9151 *	1.8449–4.6059
**Street tree count (100 m buffer)**					
Adjusted	1.0004	0.9994–1.0014	Adjusted	1.0004	0.9988–1.0019
Unadjusted	0.9977 *	0.9967–0.9987	Unadjusted	0.9993	0.9977–1.0009
**Street tree count (250 m buffer)**					
Adjusted	1.0001	0.9998–1.0003	Adjusted	1.0001	0.9997–1.0005
Unadjusted	0.9993 *	0.9990–0.9995	Unadjusted	0.9997	0.9994–1.0001
**Street tree count (500 m buffer)**					
Adjusted	1	0.9999–1.0001	Adjusted	1	0.9998–1.0001
Unadjusted	0.9997 *	0.9997–0.9998	Unadjusted	0.9998 *	0.9997–0.9999

* Denotes significance. The adjusted models control for maternal age, race, region of birth, education, Medicaid status, marital status, tobacco use during pregnancy, and alcohol use during pregnancy; the infant’s gestational age, sex, and season of birth; the distance to industrial land use; the distance to the nearest major road; neighborhood deprivation; population density; access to major green space; and access to publicly accessible waterfront areas.

## Supplementary Materials

The following are available online at www.mdpi.com/1660-4601/14/7/771/s1, Table S1: Unadjusted mixed-effects logistic regression results for access to green spaces, Table S2: Unadjusted mixed-effects logistic regression results for waterfront access.
