# Supplementary material for: Green Streets: Urban Green and Birth Outcomes
Source: Int J Environ Res Public Health. 2017 Jul 13;14(7):771. doi: 10.3390/ijerph14070771 (PMC5551209; doi:10.3390/ijerph14070771)
Supplement: Supplementary file 1 [file ijerph-14-00771-s001.pdf]

Supplementary Materials: Green Streets: Urban Green and Birth Outcomes

Kathryn Abelt * and Sara McLafferty

Table S1. Unadjusted mixed-effects logistic regression results for access to green spaces.

Study Population		Deprived Tracts	
**Term birthweight**			
**Coef.**	**95% CI**	**Coef.**	**95% CI**
−11.5586 *	−21.9007–−1.2166	−26.9744 *	−43.0978–−10.8510
**Odds of term low birthweight**			
**OR**	**95% CI**	**OR**	**95% CI**
1.0682	0.9506–1.2003	1.1942	0.9908–1.4394
**Odds of preterm birth**			
**OR**	**95% CI**	**OR**	**95% CI**
1.0987 *	1.0242–1.1786	1.1503 *	1.0317–1.2825
**Odds of small for gestational age (SGA)**			
**OR**	**95% CI**	**OR**	**95% CI**
1.0538	0.9888–1.1230	1.1510 *	1.0438–1.2692

* Denotes significance.

Table S2. Unadjusted mixed-effects logistic regression results for waterfront access.

Study Population		Deprived Tracts	
**Term birthweight**			
**Coef.**	**95% CI**	**Coef.**	**95% CI**
5.7085	−2.9908–14.4079	9.0286	−2.6690–20.7262
**Odds of term low birthweight**			
**OR**	**95% CI**	**OR**	**95% CI**
0.9112	0.8268–1.0042	0.9623	0.8461–1.0945
**Odds of preterm birth**			
**OR**	**95% CI**	**OR**	**95% CI**
1.009	0.9522–1.0691	0.9717	0.8996–1.0495
**Odds of SGA**			
**OR**	**95% CI**	**OR**	**95% CI**
0.9862	0.9359–1.0392	1.011	0.9449–1.0817

© 2017 by the authors; licensee MDPI, Basel, Switzerland. This article is an open access article distributed under the terms and conditions of the Creative Commons by Attribution (CC-BY) license (http://creativecommons.org/licenses/by/4.0/).
